# Factors influencing the length of postgraduate training and motives for choosing general practice as a specialty. Results of a cross-sectional study of general practitioners after completion of the specialist examination

**DOI:** 10.3205/zma001722

**Published:** 2024-11-15

**Authors:** Martin Fink, Ida Lotter, Monika Sennekamp

**Affiliations:** 1 Goethe University Frankfurt, Institute of General Practice, Frankfurt/Main, Germany

**Keywords:** medical education, postgraduate training, general practice, length of postgraduate training, time of decision, general practitioner shortage

## Abstract

**Background:**

The attractiveness of general practice (GP) is increasing, as is evident in its growing popularity among students and the sharp rise in specialist certifications. However, in view of the future challenges at the GP level of care, there is a shortage of young doctors. It would thus be desirable if postgraduate training could be completed quickly, particularly in this area.

**Objective:**

The aim of this article is to improve the state of knowledge of the actual length of postgraduate training in general practice and to identify the motives for choosing this specialty and possible factors influencing postgraduate training length.

**Project description:**

The cross-sectional study conducted of general practitioners (GPs) after completion of the medical specialist examination (survey period January 2020 to September 2023, population 530 persons, response rate 50%, n=265) examines the decision-making processes and the individual course of postgraduate training as pursued by newly certified general practitioners (Fachärzt*innen für Allgemeinmedizin - FÄ AM) in Hesse, Germany.

**Results:**

Excluding lateral entrants (Quereinsteiger*innen), the respondents’ length of postgraduate training was 8.88 years on average (SD=3.97, median 7.75 years, Q1=6.0, Q3=10.0). The main reasons stated for choosing the specialty were personal role models and experiences made while studying. The descriptive analysis of the data collected indicates that an early choice of specialization could be associated with a shorter length of postgraduate training.

**Conclusion:**

Positive experiences during medical studies can contribute to a doctor’s decision in favor of a specialty and may possibly be a factor in shortening the subsequent length of postgraduate training. From a healthcare perspective, medical education and postgraduate training should therefore be more closely linked so that those starting postgraduate training in general practice have a firm specialty preference in mind more frequently in future.

## Zusammenfassung

**Hintergrund:**

Die Attraktivität der Allgemeinmedizin nimmt, wie der zunehmende Zuspruch aus der Studierendenschaft und die stark angestiegenen Facharztanerkennungen verdeutlichen, zu. Mit Blick auf die zukünftigen Herausforderungen auf der hausärztlichen Versorgungsebene zeigt sich dennoch ein Nachwuchsmangel. Daher wäre es wünschenswert, wenn die Weiterbildung insbesondere in diesem Bereich zügig absolviert werden könnte.

**Zielsetzung:**

Die Zielsetzung des Beitrags ist es, eine Verbesserung des Kenntnisstands zur realen Dauer der Weiterbildung Allgemeinmedizin zu leisten, sowie Motive für die Fachrichtungswahl und mögliche Einflussfaktoren auf die Weiterbildungsdauer zu benennen.

**Projektbeschreibung:**

Die durchgeführte Querschnittsstudie mit Allgemeinärzt*innen nach Abschluss der Facharztprüfung (Erhebungszeitraum Januar 2020 bis September 2023, Grundgesamtheit 530 Personen, response rate 50%, n=265) untersucht die Entscheidungsprozesse und Weiterbildungsverläufe der frisch examinierten Fachärzt*innen für Allgemeinmedizin (FÄ AM) in Hessen.

**Ergebnisse:**

Die Weiterbildungsdauer der Befragten betrug, ohne Berücksichtigung von Quereinsteiger*innen, im Mittel 8,88 Jahre (SD=3,97; Median 7,75 Jahre, Q1=6,0, Q3=10,0). Als Gründe für die Wahl der Fachrichtung wurden vorrangig persönliche Vorbilder und Erfahrungen aus dem Studium genannt. Die deskriptive Analyse der erhobenen Daten weisen darauf hin, dass eine frühzeitige Fachrichtungswahl mit einer kürzeren Weiterbildungsdauer assoziiert sein könnte.

**Schlussfolgerung:**

Positive Erfahrungen im Studium können zur Entscheidung für eine Fachrichtung beitragen und sind womöglich geeignet, die spätere Weiterbildungsdauer zu verkürzen. Aus Versorgungsperspektive sollten daher Aus- und Weiterbildung stärker zusammen gedacht werden, so dass die Weiterbildung Allgemeinmedizin perspektivisch häufiger mit gefestigtem Fachrichtungswunsch begonnen wird.

## Introduction

### Background

In some specialist areas, such as general practice and surgery, there is a shortage of young doctors [1]. With regard to general practice, which is key to primary care, the German Council of Economic Experts for the Assessment of Developments in the Healthcare System (*SVR Gesundhei*t) already pointed out in its 2014 report that, on average, only one in two GPs who cease to practice because of retirement can find a successor [2].

General practice has in fact become increasingly popular as a result of various efforts in recent years, as can be seen in the National Association of Statutory Health Insurance Physicians (*Kassenärztliche Bundesvereinigung – KBV*) occupational monitoring [1], [3] and the significant increase in the number of medical specialist graduates [4], [5]. However, this development, which is positive in terms of ensuring nationwide outpatient care, is countered by the increasing complexity of medicine, the growing need for medical care as society ages, and the demographic depletion of rural areas [6], [7]. What is more, the general practitioners participating in statutory healthcare in Germany are structurally over-aged [8] in many places and will therefore not be available for patient care for much longer.

In view of these facts, it would be desirable if postgraduate training in this area in particular were completed quickly so that trainees (*ÄiW*) are available earlier for autonomous patient care. We take this as an opportunity to look at the length of postgraduate training in general practice, the motives for choosing this specialty and possible factors influencing postgraduate training length.

### State of research: What we know about the time needed to complete medical school and postgraduate medical training in general practice in Germany

The data available on the duration of medical studies, i.e. basic medical “education”, indicates hardly any potential for optimization: The drop-out rate of medical students is only around 10%, which is well below the overall average for university drop-outs [9]. According to the Federal Statistical Office of Germany (*Statistisches Bundesamt*), the median total time spent to complete a university degree is 13.9 semesters [10], [11]. Over 60% of students complete their studies within the standard time to degree, with over 88% of medical students only requiring a maximum of two semesters more than the standard time to degree [12], [13]. If one also takes into account that medical students often earn a doctorate while completing their basic medical studies, these are very good figures [14].

However, this cannot be said about medical “postgraduate training”, looking at the length of postgraduate training or the related data available: The structures of university training and postgraduate specialist training in hospitals and practices differ significantly. While the universities carry out various monitoring activities, the results of which automatically enter into official statistics, the actual length of postgraduate specialist training – i.e. the period between being awarded a license to practice medicine (*Approbation*) and full board certification by passing the specialist examination (*Facharztprüfung*) – is apparently not systematically recorded, or at least not communicated, by either the medical associations or German states’ statistical offices [15]. One should also bear in mind that, unlike the standard time to degree at universities, the time specifications in the regulations on postgraduate training requirements (*Weiterbildungsordnungen – WBO*) of the federal states’ medical associations (*Landesärztekammern – LAEK*), which are based on the sample regulation on postgraduate training requirements of the German Medical Association (*Musterweiterbildungsordnung der Bundesärztekammer – MWBO*), are expressly considered “minimum times” [15].

### What is the state of knowledge about the actual length of postgraduate specialist training?

A study published by Huenges et al. in 2010, based on 123 responses from a survey of 438 physicians completing their postgraduate training in various specialties from Westphalia-Lippe, cites an expected average length of postgraduate training of 9.5 years (median 8 years) [16]. Although this is a prospective survey conducted regionally, it provides the best evidence to date on the actual duration of postgraduate specialist training [2]. A study published in 2018 by van den Bussche et al. on the length of postgraduate training is also based on prognostic information supplied by physicians in postgraduate training [15]. In this longitudinal study, a cohort that completed their mandatory resident final year (*Praktisches Jahr – PJ*) in 2008/2009 was surveyed by post at seven different assessment points. Of the doctors in their fifth year of postgraduate training, 58% (n=317) stated that they needed “(significantly) longer” than the minimum period given for postgraduate training. This publication also contains references to other studies according to which the minimum postgraduate training period is often exceeded, including in surgery [17], [18] and other specialties ([19] as cited in [15], [20]). There are two further studies with a specific focus on postgraduate training in general practice: A retrospective analysis of the database of the Westphalia-Lippe Medical Association published in 2015, of almost 4,000 doctors who obtained full board certification as general practitioners between 1992 and 2012, paints a more drastic picture of the length of postgraduate training. This, operationalized as the period between the first professional license and full board certification as specialist, is 10.7 years on average according to this data (median 8.6 years) [21]. Broermann et al. surveyed graduates that passed the specialist examination in general practice in Hesse in the period from 2013 to 2016. The respondents in that survey reported an average of just under 8 years and 8 months as the length of postgraduate training, defined as the period between licensure and passing the specialist examination [22].

The various studies indicate an average length of postgraduate training for the specialty of general practice of between approximately 8.7 and 10.7 years, which roughly corresponds to twice the stipulated minimum length of postgraduate training. Gender [21], [22], parenthood [15], [16], [21], [22], also (resultant) part-time work [16], [22], non-creditable activities and phases of job/training application [16] or, stated more generally, “official” and “personal” reasons [20] are cited as (possible) reasons for the length of postgraduate training.

The studies by Huenges et al. [16] and Broermann et al. [22] also suggest that the decision to pursue postgraduate training in the general practice specialty is usually only made during postgraduate training and thus relatively late [16].

Although the fact that the required minimum postgraduate training time is often significantly exceeded is not a phenomenon specific to general practice ([17], [18], [19] as cited in [15], [20]), it is of particular importance in general practice given that outpatient primary care is already overburdened in many places as outlined above. For this reason, the actors involved are attempting to manage outpatient primary care supply capacities by employing a wide range of measures, from rural doctor quotas, income guarantees, and establishment -of-practice premiums, to waiving the residency requirement [23]. For specifically focusing on postgraduate training in general practice, the following deserve mention first and foremost: the competency centers set up for postgraduate medical training (*Kompetenzzentren Weiterbildung – KW*), which contribute to improving the quality and efficiency of general practice postgraduate training [https://www.sozialgesetzbuch-sgb.de/sgbv/75a.html] [24]. They do so by offering seminar and mentoring programs for general practitioners in postgraduate training, and train-the-trainer seminars for those providing postgraduate training. Also deserving particular mention in this sense are the postgraduate training associations (*Weiterbildungsverbünde*), as well as the regional Associations of Statutory Health Insurance Physicians (*Kassenärztliche Vereinigungen*) for their financial support of the outpatient postgraduate training track.

All in all, one has to agree even today with van den Bussche’s finding that “[t]he data available on postgraduate medical training [...] [is] so extremely deficient that not even the simplest questions on this complex of topics can be answered reliably” [15].

### Central question

With a view to the state of research on the length of postgraduate training in general practice and the underlying facts in this regard, we have examined the question of which factors may influence the actual length of postgraduate training, which the* SVR Gesundheit* already described as “unnecessarily long” [2] in 2014, and which motives are behind the choice of this specialty.

## Methods

### Project description

Many control options for planning outpatient primary care capacities (such as rural doctor quotas) are aimed at increasing the number of future GPs [23]. In contrast, this ongoing exploratory study “On the future of GP care: New general practitioners – who they are, how they experienced their postgraduate training and how they imagine their career in future” investigates whether an increase in healthcare capacity could be achieved with the existing number of doctors in general practice postgraduate training in Hesse, and if so, how. The study is based on a survey of the socio-structural characteristics, the paths leading to general practice, individuals’ own progress in completing postgraduate training and the future expectations of newly certified general practitioners.

### Development of the instrument

Since May 2013, the graduates who passed the general practice specialist examination in Hesse have been surveyed by means of a questionnaire on their individual progress in completing postgraduate training (see attachment 1 ) [22]. In order to understand the decision-making processes of physicians in postgraduate training to become general practitioners, the authors fundamentally revised this data collection instrument at Hesse’s Competency Center for Postgraduate Medical Training (*Kompetenzzentrum Weiterbildung Hessen - KW Hessen*) at the turn of the year 2019, involving an interdisciplinary group of experts with medical (specialist), health science, educational, psychological and sociological backgrounds, and extending the instrument to include questions on socio-structural characteristics, individual postgraduate training progression and future plans. The instrument, in use since the beginning of 2020, contains a total of around 90 questions encoded into 202 variables.

### Data collection/use of the instrument

Postgraduate general practice training in Hesse terminates with an oral exam administered by the Hesse Medical Association. All successful graduates are personally given the questionnaire we developed and a cover letter directly after passing the general practice specialist examination (see attachment 1 ). In this way, we aim to conduct a complete survey of all newly certified general practitioners in Hesse. 

Between January 2020 and September 2023, a total of 530 passes of the general practice specialist exam were recorded in Hesse. All successful graduates were personally given the questionnaire directly after passing the specialist exam (see attachment 1 ). They were requested to participate in the study, which would be on a voluntary basis. 

The data was encoded, checked for consistency and analyzed using IBM SPSS 28 with the help of the code key that was created at the time the instrument was developed.

## Results

A total of 265 completed questionnaires were submitted by October 13, 2023, i.e. three weeks after the last general practice specialist exam for the third quarter of 2023. This corresponds to a 50% response rate in accordance with the AAPOR Response Rate 2 [25]. Assessment was purely descriptive due to the small subgroups resulting from the number of cases. 

Table 1 (Tab. 1) shows that 68 of the 265 respondents (25.7%) are what are referred to as “lateral entrants” to general practice, meaning that they already hold another specialist title when they take the specialist examination in general practice. In Hesse, as in most other regional medical administration entities, such “lateral entry” into general practice is possible for specialists qualified in another area of direct patient care [26], [27].

Only two years of postgraduate training in a general practice is required for this group of entrants as opposed to the five-year minimum postgraduate training for general practice recommended by the MWBO [28]. As we define the “real” length of postgraduate training as the period between licensure and having passed the specialist examination, as do Huenges et al. [16] and Broermann et al. [22], the group of lateral entrants must be excluded from the following considerations on the length of postgraduate training.

### Length of postgraduate training

Table 2 (Tab. 2) shows the differences in the postgraduate training time by gender, which can be seen in connection with the significantly higher part-time rates for women (see table 1 (Tab. 1)). By far the most common reason given by respondents for working part-time is parenthood (stated 93 times out of the 122 responses furnished (76.2%)). Our data also shows that among those who did not (yet) have children at the time of taking the specialist examination (n=55), the male respondents (n=11) had spent an average postgraduate training time of 6.59 years, which is more than one year less than the female respondents (n=44; 7.78 years).

### Time of decision for the general practice specialty and length of time spent in postgraduate training

In order to investigate whether the time of decision for specialization in general practice has an influence on the length of time spent in postgraduate training, we divided the average postgraduate training time into decision-time subgroups (before the course of study, during the course of study, between the end of the course of study and the start of postgraduate training, and during postgraduate training), analyzed the subgroups and present the data as follows.

**Figure 1:**
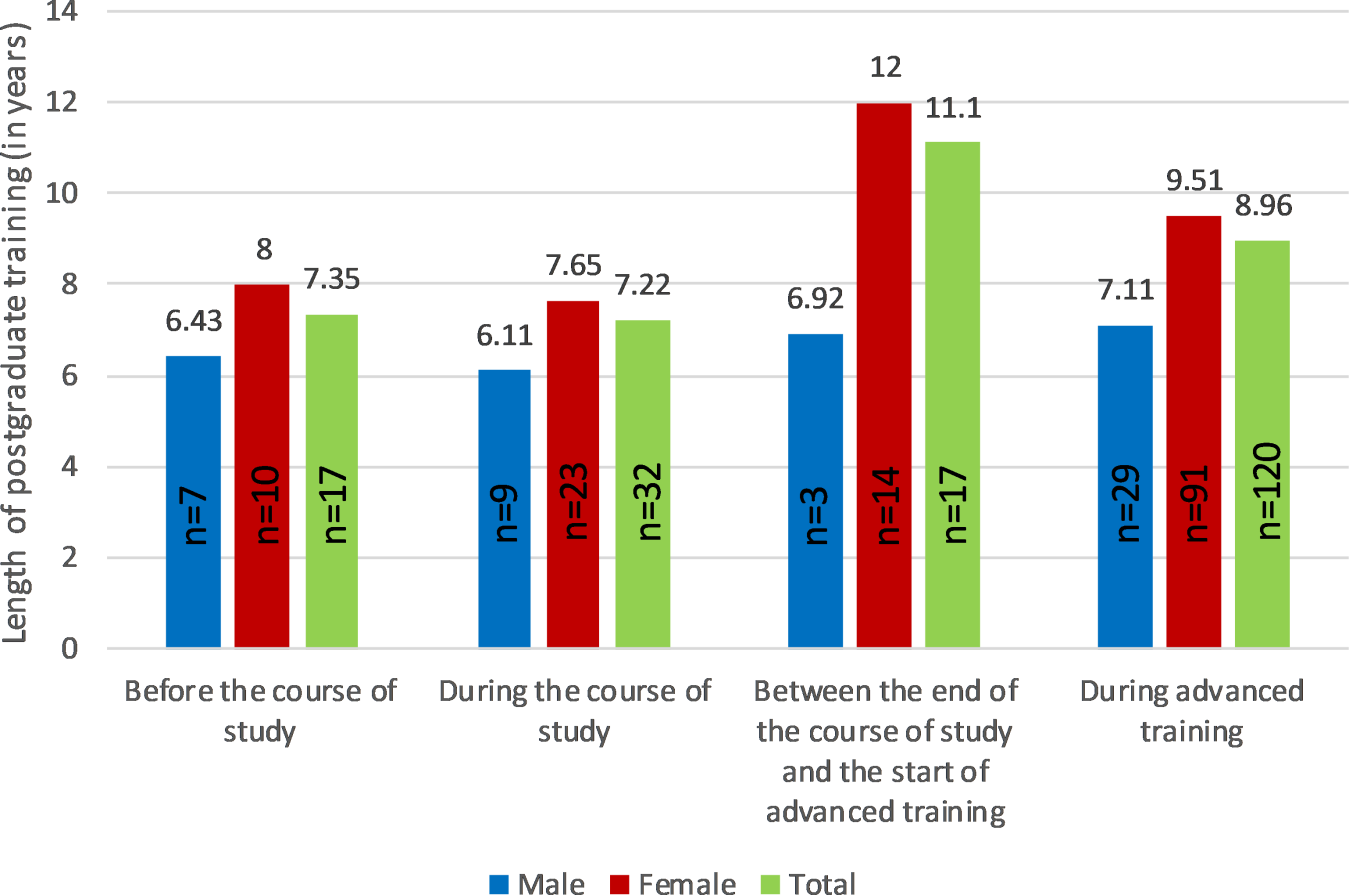
Length of postgraduate training in years, divided into subgroups by gender and time of decision in favor of postgraduate training in general practice. General practitioners after completion of the medical specialist examination were surveyed between January 2020 and September 2023, population 530 people, response rate 50%, n= 265. After excluding lateral entrals and invalid/incomplete responses n=186.

**Figure 2:**
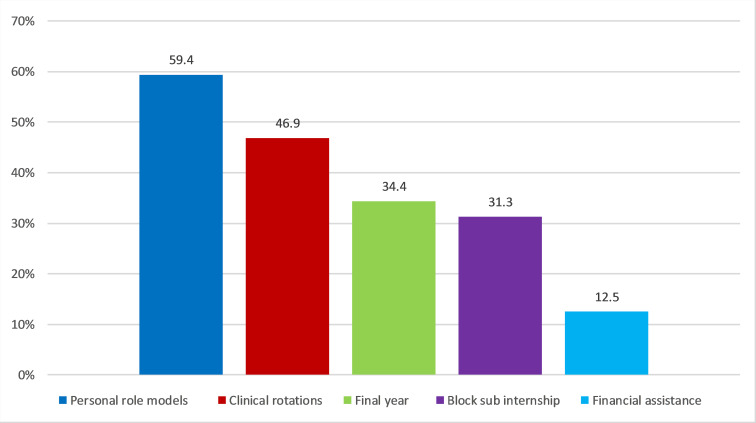
Percentage of those who completed parts of their postgraduate training on a part-time basis, divided into subgroups by gender and time of decision in favor of postgraduate training in general practice. General practitioners after completion of the medical specialist examination were surveyed between January 2020 and September 2023, population 530 people, response rate 50%, n= 265. After excluding lateral entrals and invalid/incomplete responses n=186.

Figure 1 (Fig. 1) and figure 2 (Fig. 2) show that, especially for female doctors, the part-time rate is higher and the postgraduate training period (therefore) longer if the decision to specialize was not made before or during the course of study, but at a later date after the end of the course of study.

### Reasons for choosing general practice as a specialty

The specialists were given semi-open questions with multiple choice answers on what motivated them to complete postgraduate training in general practice. The most frequently stated reasons for postgraduate training in general practice in the small group of those who decided on this specialty before starting their studies (n=17) were personal role models (13 mentions/76.5%) and the reasons mentioned under “other” of personal interest (3 mentions/17.6%) and family influence (2 mentions/11.8%).

**Figure 3:**
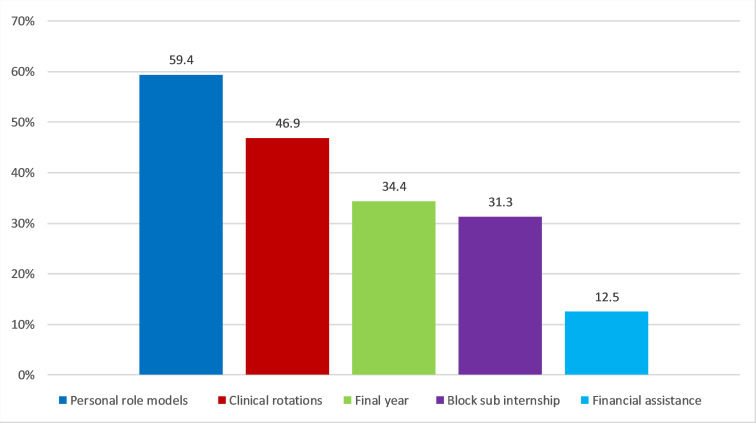
Motivation factors of those who decided to pursue postgraduate training in general practice during their studies (n=32); multiple answers possible. General practitioners after completion of the medical specialist examination were surveyed between January 2020 and September 2023, population 530 people, response rate 50%, n= 265.

The most frequently cited reasons for choosing general practice as a specialty in the group of those who decided during their studies to further train as general practitioners (n=32) were personal role models and the experiences the doctors made during their studies (clinical rotations (*Famulatur*), block sub internship (*Blockpraktikum*), final year) (see figure 3 (Fig. 3)).

For those who decided on general practice postgraduate training between the end of their studies and the start of their postgraduate training (n=17), personal role models (9 mentions/52.9%) are also the most frequently cited reason, followed by financial assistance (5 mentions/29.4%), clinical rotations (3 mentions/17.6%) and the final year (2 mentions/11.8%).

Among those who decided on general practice during their postgraduate training (n=118), the reasons most frequently mentioned in addition to personal role models (59 mentions/50%) and financial assistance (27 mentions/22.9%) were work and family life balance (19 mentions/16.1%) and working conditions (11 mentions/9.3%), both listed under “other”. In this group, the block sub internship (16 mentions/13.6%) and clinical rotations (14 mentions/11.9%) were also cited as reasons for choosing this specialty.

## Discussion

Our study shows that the average length of time spent in postgraduate training in general practice in Hesse is 8.88 years (median 7.75 years). Given the difference in the length of time spent in postgraduate training attributable to gender, and the proportion of mothers and fathers in our data, we assume that care work is unequally distributed between the genders [29], [30]. Female doctors without children also report spending more than one year longer in postgraduate training on average than their male counterparts. Furthermore, our data indicates that there could be a correlation between the point when the decision for the specialty is made and the length of postgraduate training in the sense that an early decision is associated with a shorter duration of postgraduate training. The reason most frequently cited by our respondents for choosing to specialize in general practice is personal role models. In the group of those who had already opted for general practice during their studies, the clinical rotations, the block sub internship and the final year were also most frequently cited as key reasons.

Looking at the reasons given by the newly certified GPs for their choice of postgraduate training, the contact points made during their studies appear to be well suited for promoting their own specialty. 

The average duration of postgraduate training we determined is at the lower end of the range of postgraduate training times cited in other studies [16], [21], [22]. One reason for this could be the exclusion of lateral entrants in our study based on our definition of the actual length of time spent in postgraduate training. Due to our focus on the length of postgraduate training and its resultant operationalization, the motives for lateral entrants’ choice of specialty were initially excluded from our study; for the motives of this group, see publications by Schwill et al. [31]. Our study confirms the influencing factors on postgraduate training length cited in the literature – gender, parenthood, (also resultant) part-time work. In our data, we do not find any influence of delays due to phases of job/training application. We did not collect any data on the issue of non-creditable activities.

The fact that contact with outpatient care during the course of study can be key to the choice of the future medical practice specialty is consistent with the results of the KBV career monitoring survey [https://www.kbv.de/html/1150_60415.php]: The proportion of students surveyed there who would opt for general practice rose from 7.2% (during the preclinical phase) to 10.9% after the final year [1].

This is the only current study known to the authors to systematically examine the decision time for the choice of specialty as a factor influencing duration of postgraduate training and one with high penetration of the population; thus, there are no indications – recognizable to the authors – that self-selection of participation or non-participation in the study by the newly certified specialists could have led to any systematic distortion of the response. Depending on operationalization or available data, lateral entrants may not be adequately taken into account in the reported durations of postgraduate training. In our view, the mere description of the actual postgraduate training time based on the information provided by 50% of all new general practitioners in Hesse is already beneficial for the discourse on the topic of postgraduate training (time).

We were able to achieve a very high response rate in our study. We attribute this primarily to the “targeted addressing” of the test subjects [32], [33]. Unfortunately, due to the overall small number of cases – and the resultant small subgroups – as well as the multicollinearity of the variables of gender, parenthood and part-time work described as influencing factors on postgraduate training time, the prerequisites for the application of suitable multivariate statistical methods for analysis are not (yet) met. Therefore, for example, the possibility of a correlation between the motives for the choice of specialty and the postgraduate training duration could not (yet) be adequately investigated.

In principle, better data quality on the actual duration of postgraduate training (in the sense of a complete survey) would be possible if based on the states’ medical association data, which could, for example, be automatically generated in the introduction of electronic logbooks. However, even if this data were available, further scientific studies would be required to investigate the reasons for the choice of specialty and the characteristics of the postgraduate training period.

Personal role models and experiences during studies can contribute significantly to which specialty/specialties students perceive as interesting. Our data also suggests that students with a clear postgraduate training specialty in mind complete postgraduate training relatively quickly.

Particularly in specialties with problems in recruiting young doctors, medical education and postgraduate training should be (even) more closely linked and the transition as seamless as possible. On the one hand, the relatively “open” regulation on postgraduate training requirements (*Weiterbildungsordnung – WBO*) in general practice [34] makes it possible to gain (creditable) experience in (very) different areas, which can have a positive effect on the quality of care. On the other hand, job changes during the course of a doctor’s postgraduate training or the clinical phase of postgraduate training may make it more difficult for them to identify with general practice. This can well be addressed by the Competency Center for Postgraduate Medical Training offerings, especially if the postgraduate training objective has already been determined at an early stage. For those who wish to resume their postgraduate training after a longer break, specific re-entry offerings can lend valuable support.

## Conclusions

Positive experiences during medical studies can contribute to a doctor’s decision in favor of a specialty and may possibly be a factor in shortening the subsequent length of time spent in postgraduate training. From a healthcare perspective, medical education and postgraduate training should therefore be more closely linked, particularly in specialties with problems in recruiting young doctors, so that physicians start their postgraduate training with a firm specialization in mind more frequently. Further studies on the postgraduate training phase are needed to investigate the reasons for the choice of specialty and the characteristics of the postgraduate training period.

## Notes

### Funding

The Hesse Competency Center for Postgraduate Medical Training is financially supported by the Hesse Ministry of Social Affairs and Integration (since January 2024 Hesse Ministry of Family Affairs, Senior Citizens, Sport, Health and Care) (funding code 08 06 no. 46) and receives funding in accordance with section 75a (7) of the Fifth Volume of the German Social Security Code (Sozialgesetzbuch, Fünftes Buch – SGB V). We would like to express our thanks for this funding. 

### Authors’ ORCIDs

- Martin Fink: [0000-0002-8948-5889]
- Ida Lotter: [0009-0000-8810-3351]
- Monika Sennekamp: [0009-0009-5270-4775]

## Acknowledgements

The authors would like to thank the Hesse Medical Association for the good cooperation and Elias Linke and Nele Wilters for their support in data collection and entry. We also thank Anna McSherry for the professional translation of the article in English. 

## Competing interests

The authors declare that they have no competing interests. 

## Supplementary Material

Questionnaire

## Figures and Tables

**Table 1:**
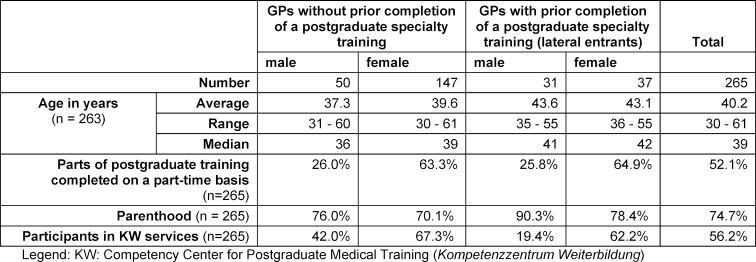
Data basis

**Table 2:**
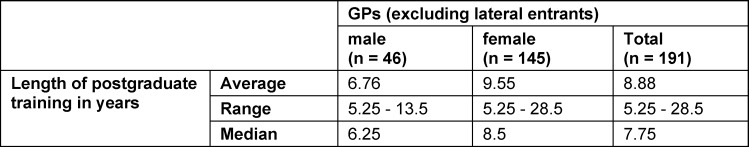
Length of postgraduate training of general practitioners
