# Supplementary material for: Factors influencing the length of postgraduate training and motives for choosing general practice as a specialty. Results of a cross-sectional study of general practitioners after completion of the specialist examination
Source: GMS J Med Educ. 2024 Nov 15;41(5):Doc67. doi: 10.3205/zma001722 (PMC11656183; doi:10.3205/zma001722)
Supplement: Questionnaire [file JME-41-67-s-001.pdf]

Attachment 1: Questionnaire

Attachment 1 to Fink M, Lotter I, Sennekamp M. *Factors influencing the length of postgraduate training and motives for choosing general practice as a specialty. Results of a cross-sectional study of general practitioners after completion of the specialist examination.* GMS J Med Educ. 2024;41(5):Doc67. DOI: 10.3205/zma001722

The English version is a translation for informational purposes only of the original German-language instrument.

Please create your personal code below.

A) **first** letter of your mother's first name (e.g. **B**eate → B)

B) **first** letter of the first place you studied medicine (e.g. **L**eizpig → L)

C) & D) mother's date of birth (**DD** MM YYYY) (e.g. **03**.05.1960 → 0 3)

E) **first** letter of your place of birth (e.g. **B**ad Hersfeld → B)

5-digit code (please fill in)

A	B	C	D	E

If you cannot answer one or more of these questions, please put an "X" in the corresponding place.

I. General information

1. Your year of birth: ☐☐☐☐

2. Your gender: ☐ female ☐ male ☐ other

3. Your nationality: ____________________________________

4. What is the population of the main place in which you grew up?
☐ less than 5,000 ☐ between 5,001 and 20,000 ☐ between 20,001 and 50,000
☐ between 50,001 and 100,000 ☐ over 100,000 ☐ I don't know/no answer

5. Your marital status:
☐ married/registered civil partnership ☐ in partnership ☐ divorced ☐ single ☐ widowed

→ If you have a partner, in what year were they born? ☐☐☐☐

6. What is the population of the main place in which your partner grew up?
☐ I do not have a partner
☐ less than 5,000 ☐ between 5,001 and 20,000 ☐ between 20,001 and 50,000
☐ between 50,001 and 100,000 ☐ over 100,000 ☐ I don't know/no answer

7. Do you live with your partner in the same household?
☐ I do not have a partner ☐ no ☐ yes

8. What is your partner's profession? Please describe the occupation as precisely as possible:

____

9. Based on your personal life planning, who in your partnership do you expect to be the main earner in the long term?
☐ I do not have a partner
☐ me ☐ I am likely to be ☐ each likely to contribute equally ☐ my partner is likely to be ☐ my partner

10. Do you have children? ☐ no ☐ yes If yes:
a. How many children do you have? ☐
b. year of birth of your youngest child: ☐☐☐☐
c. year of birth of your oldest child: ☐☐☐☐

11. Had you already taken up vocational training/another course of study prior to studying medicine?
☐ yes, completed it, namely: ____
☐ yes, but did **not** complete it, namely: ______________________________________
☐ no

12. Quarter and year you were awarded license to practice medicine (*Approbation*): ☐ ☐☐☐☐

13. Place of your license to practice medicine ☐ Germany ☐ outside Germany, in: ______________________

14. Quarter and year you started practicing medicine after obtaining your license: ☐ ☐☐☐☐

15. Quarter and year you took the specialist examination in general practice: ☐ ☐☐☐☐

a. If you had already completed specialist training before, in what specialty? ____________________________

b. If applicable, the quarter and year of any previous specialist examination: ☐ ☐☐☐☐

16. How well do the following statements describe your personality?

I see myself as someone who…

	(1) Disagree strongly	(2) Disagree a little	(3) Neither agree nor disagree	(4) Agree a little	(5) Agree strongly
(1) ... is reserved	☐	☐	☐	☐	☐
(2) ... is generally trusting	☐	☐	☐	☐	☐
(3) ... tends to be lazy	☐	☐	☐	☐	☐
(4) ... is relaxed, handles stress well	☐	☐	☐	☐	☐
(5) ... has few artistic interests	☐	☐	☐	☐	☐
(6) ... is outgoing, sociable	☐	☐	☐	☐	☐
(7) ... tends to find fault with others	☐	☐	☐	☐	☐
(8) ... does a thorough job	☐	☐	☐	☐	☐
(9) ...gets nervous easily	☐	☐	☐	☐	☐
(10) ... has an active imagination	☐	☐	☐	☐	☐

II. Information on individual postgraduate training

17. Are you familiar with the services of Hesse's Competency Center for Postgraduate Medical Training (*Kompetenzzentrum Weiterbildung Hessen* - KW Hessen) for physicians in postgraduate general practice training?

☐ no ☐ yes → **If yes:** a. Have you participated in the KW Hessen seminar program?

☐ no ☐ yes, namely __________ years, most recently: __________

b. Have you participated in the KW Hessen mentoring program?

☐ no ☐ yes, namely __________ years, most recently: __________

c. How did you hear about the KW Hessen? ____

____

d. What (other) support services would you like to have been offered by KW Hessen?

____

____

18. Have you taken advantage of services by the Hesse Association of Statutory Health Insurance Physicians (*Kassenärztliche Vereinigung Hessen* - KVH) for physicians in postgraduate general practice training?

☐ no ☐ yes → If yes, which of these services?

☐ financial assistance for the clinical rotations ☐ summer and winter school

☐ financial assistance for postgraduate training ☐ 'Doc's Camp'

☐ financial assistance for the final year

☞

19. What made you decide to pursue postgraduate training in general practice? (multiple answers possible)

☐ clinical rotations ☐ 'Doc's Camp' ☐ curriculum focus on general practice

☐ block sub internship ☐ 'Landpartie' edu. program ☐ financial assistance from the KVH

☐ Final year ☐ 'Praxistrack' ☐ KW Hessen's introductory weekend

☐ summer and winter school ☐ personal role models ☐ other:___________________________________

20. I received financial assistance from the KVH for the following program: (multiple answers possible)

☐ clinical rotations ☐ final year ☐ outpatient part of postgraduate training

☐ none ☐ I don't know/no answer

21. How important was the KVH financial assistance for you? very important ☐ ☐ ☐ ☐ ☐ absolutely not important

22. Did you complete your entire postgraduate training in Hesse? ☐ yes ☐ no

→ If no: In what other federal German state/other country did you complete parts of your postgraduate training?

____

23. How did you find your jobs? (multiple answers possible)

☐ job exchange of the Coordination Office for Postgraduate Education in General Practice Hesse (*Koordinierungsstelle Weiterbildung Allgemeinmedizin Hessen*)

☐ unsolicited application ☐ Hessenkarte (postgraduate training networks) of the Coordination Office

☐ personal contacts ☐ Hesse's Competency Center for Postgraduate Medical Training

☐ by other means: ____

24. How often did you apply for a job during your postgraduate training in general practice? ☐☐ - times

25. Did you openly communicate the aim of your postgraduate training (to become a GP), particularly in the clinical part of your postgraduate training?

☐ yes ☐ no → If no: Why not? ____

26. Did you have one or more <u>undesired</u> interruptions of at least one month in your postgraduate training?

☐ yes, a total of _______ months. The reasons for this were ____

☐ no

27. Did you have one or more <u>desired</u> interruptions of at least one month in your postgraduate training?

☐ yes, a total of _______ months. The reasons for this were ____

☐ no

28. Did you complete parts of your postgraduate training on a part-time-basis?

☐ no ☐ yes → If yes: What part(s)?

____

→ Please state the reasons for this: _____________________________________

→ Under what conditions would a full-time job have been an option for you?____

____

29.1 How long did you work in inpatient care during your postgraduate training in general practice? (estimate if necessary)

_________________ years

29.2 How long did you work in outpatient care during your postgraduate training in general practice? (estimate if necessary)

_________________ years

30. When did you decide to complete postgraduate training in general practice?

☐ before the course of studying ☐ during the course of study ☐ between the end of the course of study and the start of the postgraduate training

☐ during the postgraduate training ☐ after completing other postgraduate training

→ If "during the postgraduate training" or "after completing other postgraduate training":

a. Please explain:

____

____

____

b. Please state your original postgraduate training objective:

____

31. Please state for us the quarter and year in which you **decided** to complete your postgraduate training in general practice.

☐ ☐☐☐☐

32. Please state for us the quarter and year in which you actually **started** to complete your postgraduate training in general practice.

☐ ☐☐☐☐

33. What were the main reasons to become a general practitioner?

____

____

34. What do you consider obstacles to choosing general practice?

____

____

35. Did a break in pursuing your career (e.g. parental leave) play a role in your decision to specialize in general practice?

☐ yes ☐ no

☞

36.1 How many physicians in postgraduate general practice training (ÄiW) or “new” general practitioners do you personally know? (estimate if necessary) ____________

36.2 How many of them participate or have participated in the KW Hessen’s services?

(estimate if necessary) ____________

37. How do you rate the image of general practice...in your own opinion? very low ☐ ☐ ☐ ☐ ☐ very high

… in society at large? very low ☐ ☐ ☐ ☐ ☐ very high

…among your colleagues in other specialties? very low ☐ ☐ ☐ ☐ ☐ very high

38. In your opinion, what degree of organizational effort did you have to put into organizing the postgraduate training phases to be completed? very low ☐ ☐ ☐ ☐ ☐ very high

39. From whom did you receive detailed and competent advice regarding your postgraduate training? (multiple answers possible)

☐ federal states’ medical associations (LÄK)
☐ university instructors
☐ Association of Statutory Health Insurance Physicians (KVH)
☐ KW Hessen
☐ GPs
☐ other sources: ____________________
☐ friends
☐ Coordination Office
☐ from no one

40. To what extent do you agree with the following statements about postgraduate training in general practice?	fully agree	agree	tend to agree	tend to disagree	do not agree	do not agree at all	no answer
(1) I think that postgraduate training in general practice is well structured.	☐	☐	☐	☐	☐	☐	☐
(2) I think that postgraduate training in general practice in the form I experienced it permits a good work-family life balance.							
(a) clinic	☐	☐	☐	☐	☐	☐	☐
(b) practice	☐	☐	☐	☐	☐	☐	☐
(3) I felt well networked with other physicians in postgraduate training (ÄiW).	☐	☐	☐	☐	☐	☐	☐
(4) My respective employer released me from my work for my desired further training sessions.							
(a) clinic	☐	☐	☐	☐	☐	☐	☐
(b) practice	☐	☐	☐	☐	☐	☐	☐
(5) My respective employer assumed the costs for participation in KW Hessen’s service offering.	☐	☐	☐	☐	☐	☐	☐
(6) I consider a structured seminar program with specialty-relevant topics, especially for ÄiW, to be important.	☐	☐	☐	☐	☐	☐	☐
(7) I consider an organized mentoring program, in which, for example, a group of ÄiW meets with a mentor at regular intervals, to be important.	☐	☐	☐	☐	☐	☐	☐

(8) I consider regular feedback from the trainer to be important.	☐	☐	☐	☐	☐	☐	☐
(9) I had the opportunity to give feedback to my employer.	☐	☐	☐	☐	☐	☐	☐
(10) I consider the opportunity to give feedback to my employer to be important.	☐	☐	☐	☐	☐	☐	☐
(11) Overall, I was very satisfied with my postgraduate training.	☐	☐	☐	☐	☐	☐	☐

41. How often did feedback conversations with the trainer take place during your postgraduate training?

clinic: ____ time(s) per year ☐ other: ____________________

practice: ____ time(s) per year ☐ other: ____________________

42. I was able to make good use of the feedback I received from my trainer for my postgraduate medical training.

☐ yes ☐ no ☐ partially → If "partially", please explain: ____________________

____

43. Did you take part in an organized mentoring program outside the KW Hessen, in which, for example, an experienced specialist mentored one or more physicians in postgraduate training?

→ If yes, the name of the program:

☐ no ☐ yes ____________________

→ If yes, was the program helpful? not helpful ☐ ☐ ☐ ☐ ☐ ☐ very helpful

44. Did you complete your postgraduate training in a postgraduate training association (*Weiterbildungsverbund*), in which practices and clinics jointly coordinated your postgraduate training?

☐ no ☐ yes ☐ partially

→ If "yes" or "partially": How did you find out about the postgraduate training association?

☐ advice from the Coordination Office ☐ Hessenkarte of the Coordination Office

☐ job exchange of the Coordination Office ☐ advice from KW Hessen

☐ personal contacts ☐ other ____________________

→ If "yes" or "partially": Would you recommend postgraduate training in a postgraduate training association?

☐ yes ☐ no ☐ I don't know

45. How would you rate the ratio of workload to remuneration during your postgraduate training?

clinic: very good ☐ ☐ ☐ ☐ ☐ ☐ very poor

practice: very good ☐ ☐ ☐ ☐ ☐ ☐ very poor

46. How important was the following to you when choosing general practice as a specialty?	very important	important	not so important	unimportant
(1) to have regular working hours	☐	☐	☐	☐
(2) to be able to organize your working hours flexibly (for example, to work part-time)	☐	☐	☐	☐

☞

	very important	important	not so important	unimportant
(3) to have good earnings potential	☐	☐	☐	☐
(4) to have good career opportunities	☐	☐	☐	☐
(5) to have a good work-family life balance	☐	☐	☐	☐
(6) to be very familiar with patients' living conditions besides just knowing their medical history	☐	☐	☐	☐
(7) to be able to treat as broad a range of illnesses as possible in this profession	☐	☐	☐	☐
(8) to work in a team with different types of specialists in this profession	☐	☐	☐	☐
(9) to work in a team with colleagues from various other healthcare professions	☐	☐	☐	☐
(10) to work in your own practice	☐	☐	☐	☐
(11) to be involved with research topics and studies	☐	☐	☐	☐
(12) the compatibility of your workplace with your partner's working conditions	☐	☐	☐	☐
(13) to work in a team with other practitioners of the same specialty	☐	☐	☐	☐
(14) to have the workplace close to home	☐	☐	☐	☐
(15) to be able to work autonomously	☐	☐	☐	☐
(16) a good range of further and postgraduate training courses on offer	☐	☐	☐	☐
(17) company childcare	☐	☐	☐	☐

47. Where do you see yourself working in **2** years' time?

☐ own practice ☐ emp. in a practice/outpatient HC center ☐ clinic
☐ research ☐ employment in the private sector

☐ other ____

48. How many hours per week would you like to be working in **2** years' time? ☐☐ hours

49. Where do you see yourself working in **10** years' time?

☐ own practice ☐ emp. in a practice/outpatient HC center ☐ clinic
☐ research activity ☐ employment in the private sector

☐ other ____

50. How many hours per week would you like to be working in **10** years' time? ☐☐ hours

51. With a view to a possible future practice of your own: Which would be your preferred path?

☐ setting up a new practice ☐ taking over an existing practice or part of a practice

☐ other: ____

52. If you could imagine settling down in a practice of your own: Which form of practice would you prefer?

☐ single practice ☐ joint practice ☐ group practice by partnership agreement (*Berufsausübungsgemeinschaft* - BAG, formerly *Gemeinschaftspraxis*)

☐ outpatient healthcare center (MVZ) ☐ other:

53. If you cannot imagine settling down to practice: What are your reasons for not wishing to do so?

____

____

54. Do you feel well prepared to set up your own practice?

☐ yes ☐ no → If no, please explain: ____________________________________

____

55. Would you be in favor of the KW Hessen offering interested physicians the opportunity to participate in coaching to accompany handover and takeover of a practice?

☐ yes ☐ no

56. Would you yourself take advantage of such a coaching offer by KW Hessen to accompany takeover of a practice?

definitely: yes ☐ ☐ ☐ ☐ ☐ ☐ definitely: no

57. What would be the ideal population of your future place of work (workplace does not have to be the place of residence)?

☐ less than 5,000 ☐ between 5,001 and 20,000 ☐ between 20,001 and 50,000
☐ between 50,001 and 100,000 ☐ over 100,000 ☐ I don't know/no answer

58. Where do you want to work in the future?

☐ in Hesse ☐ in another German state → If yes, in: ____________________________________

☐ abroad → If yes, in:____

I found this to be particularly good in the postgraduate training:

In postgraduate training in general practice, this still need improvement:

Thank you very much for participating!
